# Entering the era of precision medicine to treat amyotrophic lateral sclerosis

**DOI:** 10.1186/s13024-025-00890-5

**Published:** 2025-10-23

**Authors:** Frances Theunissen, Loren Flynn, Alfredo Iacoangeli, Ahmad Al Khleifat, Ammar Al-Chalabi, James J. Giordano, Masha Strømme, P. Anthony Akkari

**Affiliations:** 1 School of Health Science, The University of Notre Dame Australia, Fremantle, WA Australia; 2 Black Swan Biotech, Perth, WA Australia; 3 Personalised Medicine Centre, Murdoch University, Murdoch, WA Australia; 4 Perron Institute for Neurological and Translational Science, Nedlands, WA Australia; 5 Department of Biostatistics and Health Informatics, Institute of Psychiatry, Psychology and Neuroscience, King’s College London, London, UK; 6 NIHR Maudsley Biomedical Research Centre at South London and Maudsley NHS Foundation Trust and King’s College London, King’s College London, London, UK; 7 Department of Basic and Clinical Neuroscience, Maurice Wohl Clinical Neuroscience Institute, Institute of Psychiatry, Psychology and Neuroscience, King’s College London, London, UK; 8 Department of Neurology, Georgetown University Medical Centre, Washington, DC USA

**Keywords:** Personalised medicine, Antisense oligonucleotide, Genetics, Understanding heterogeneity, Long-read sequencing, Patient profiling

## Abstract

With the disease modifying therapy Qalsody (tofersen) which targets the RNA product of the *SOD1* gene, having been shown effective in amyotrophic lateral sclerosis (ALS), the present perspective seeks to explore progress towards the implementation of precision medicine principles in ALS drug development. We address the advances in our understanding of the complex genetic architecture of ALS, including the varying models of genetic contribution to disease, and the importance of understanding population genetics and genetic testing when considering patient selection for clinical studies. Additionally, we discuss the advances in long-read whole-genome sequencing technology and how this method can improve streamlined genetic testing and our understanding of the genetic heterogeneity in ALS. We highlight the recent advances in omics-data for understanding ALS patient sub-groups and how this knowledge should be applied to pre-clinical drug development in a proposed patient profiling workflow, particularly for gene targeted therapies. Finally, we summarise key ethical considerations that are pertinent to equitable care for patients, as we enter the era of precision medicine to treat ALS.

## Key points

1. Precision medicine means giving the right drug to the right patient at the right time. With the emergence of antisense oligonucleotide technologies, additional gene targeted therapies will soon enter the market for ALS.
2. The improved understanding of the complex genetic architecture of ALS and pathophysiological mechanisms involved in disease has increased the number of validated drug targets for ALS.
3. Long-read sequencing will revolutionise our understanding of ALS genetics through the comprehensive mapping of genetic variation. Long read sequencing has enabled the complete sequence of a human genome and subsequently, the first human global and population-specific pan-genome references, adding hundreds of millions of bases that were not present in the previous human genome reference.
4. Leveraging omics data and machine learning methodologies will improve our understanding of both ALS phenotype and the underlying molecular heterogeneity between ALS patients, enhancing patient stratification through the detection of specific sub-groups of patients.
5. Comprehensive patient profiling through the collection of full omics data will be critical to define responder populations. A patient profiling workflow is proposed to conduct in vitro trials alongside early clinical studies as more antisense therapies begin entering the clinic.
6. Ethical strategies to consider for population targeted therapeutic development include tiered pricing models, public-private partnerships, and global distribution initiatives, all of which may be valuable for facilitating access to genetic therapies, thereby reducing inequalities in ALS care.

## Introduction

Amyotrophic lateral sclerosis (ALS), the most common form of motor neuron disease, is a life-limiting neurodegenerative disease that results in progressive muscle weakness, with the median survival being 2–4 years from disease onset [1]. While significant improvements in quality of life have been reported from ALS patients that receive multidisciplinary care [2, 3], available treatment options are limited and only show a modest survival benefit. Currently there are only three approved disease modifying therapies to treat ALS: Riluzole, Edaravone and Qalsody (tofersen, Table 1). With only three treatments contributing to varying levels of disease modification, there is considerable focus on improving both the efficacy and number of available treatment options to modify disease progression. To date, there are approximately 45 novel drugs in clinical development programs, with drug classes including small molecules, antisense oligonucleotides (ASO), adeno-associated virus gene therapies, antibody targeted treatments and anti-retroviral therapies, with the remainder of programs addressing cell therapy treatments, drug repurposing or drug reformulation of marketed compounds (see review [18]).

**Table 1:** Approved disease modifying therapies to treat ALS

Riluzole: Riluzole is an anti-excitotoxic drug that is currently considered the standard of care for the treatment of ALS [4]. In the 25 years since its approval, no other anti-excitotoxic drugs have made it to the clinic. Riluzole typically improves survival by a few months and increases the probability of surviving one additional year by 9% [5–7]. A major issue with the low efficacy of riluzole is the poor understanding of its precise mechanism of action and limited bioavailability, possibly mediated by efflux from the central nervous system (CNS), or poor capacity in crossing the blood-brain barrier and blood-spinal cord barrier [8].
Edaravone: Edaravone is an antioxidant agent that is intravenously administered for 14 days per month and was initially approved for use in Japan in 2015 and subsequently approved by the FDA in 2017. Its approval for use in treating ALS was based on a significant slowing in the rate of decline in a subset of Japanese patients as measured by the ALS functional rating scale (ALSFRS) [9]. Additional studies investigating the long-term five-year survival benefits of edaravone are still ongoing [10], with a recent phase III result showing a slowing in ALSFRS decline between the treatment and control group [11]. Recently, a study investigated the long-term benefits of combination treatment of edaravone with riluzole compared to riluzole treatment only. The study failed to detect a significant change in its primary endpoint measure (ALSFRS) as well as secondary endpoints (survival probability, time to ventilation and change in disease progression), suggesting that long term edaravone treatment did not offer any slowing of disease progression compared to standard treatment with riluzole only [12]. This study utilized a European multicentre cohort [12] and it is likely that differences in population genetics influenced the underlying disease mechanisms in this group of patients, since it is known that ALS risk variants can differ between European and Asian populations [13, 14]. Like riluzole, the precise mechanism of action for edaravone is not fully established, although it has been demonstrated to reduce the effects of oxidative stress in spinal tissue after spinal cord injury in rats [15]. Again, no other drugs targeting antioxidant pathways have shown efficacy in clinical trials [4].
Qalsody: Qalsody (tofersen) is an antisense oligonucleotide developed by Ionis Pharmaceuticals and licenced to Biogen that is designed to address the gain of function toxicity related to SOD1 variants. The drug uses a 2 ´MOE/DNA chimera chemistry to induce RNase H1 mediated downregulation of SOD1 transcripts, therefore reducing the production of the SOD1 protein. The phase I/IIa multiple ascending dose study of tofersen reported reduction of SOD1 protein in cerebrospinal fluid (CSF) for all dose groups, and was accompanied with a decline in clinical measures of ALS for highest dose group (100 mg), particularly in patients with variants associated with fast progression of the disease [16]. This study noted reduction of phosphorylated neurofilament heavy (NFH) and neurofilament light (NFL) in plasma and CSF during the intervention and provided evidence for a potential biomarker of therapeutic efficacy. During the phase I/II study, a few patients exhibited increased white blood cell counts and increased protein in the CSF, however, these outcomes did not prevent tofersen from progressing to a phase III and open label extension study given the positive results of other exploratory measures (pulmonary function and muscle strength). While the phase III VALOR trial (NCT02623699) failed to meet its primary endpoint of change in ALSFRS-R [17], there were significant results reported for secondary endpoints, including reductions in the concentration of SOD1 in the CSF and NFL protein in plasma over a 28-week period, indicating positive target engagement [17]. Patients that were administered tofersen at the start of the VALOR trial displayed a smaller decline in ALSFRS-R score, the percentage of predicted slow vital capacity and handheld dynamometry score, than those that commenced treatment 28 weeks later in the open label extension phase of the study [17]. This suggests that a significant change in ALSFRS-R will be seen after an extended treatment timeframe. Additionally, the trial reported serious neurological adverse events in 7% of patients, including spinal cord myelitis (inflammation) with sensory and motor deficits, which are being further investigated in a long-term extension study, which has recently completed (NCT03070119) [17].

Within the past two years there have been several disappointing outcomes in ALS drug development including the failure of small molecule Verdipistat (a myeloperoxidase inhibitor to reduce oxidative stress) following a phase II/III trial (NCT04297683, NCT04436510) [19], the discontinuation of clinical development of several antisense oligonucleotides (ASOs) including BIIB105 targeting the gene *ATXN2* (NCT04494256) [20], and BIIB087 (NCT03626012) [21, 22] and WVE004 (NCT04931862) [23], both ASOs targeting the gene *C9orf72*. Additionally, there was also the removal by Amylyx Pharmaceuticals of Relyvrio (a combination of sodium phenylbutyrate and taurursodial targeting endoplasmic reticulum stress and mitochondrial dysfunction) from the market, following the failure of the pivotal PHOENIX phase III trial (NCT05021536) [24]. While these outcomes have been disheartening, there is promise with the result of Phase IIa IMODALS trial (NCT02059759) and MIROCALS phase IIb trial (NCT03039673) assessing the use of low dose interleukin-2 (IL-2, Aldesleukin) as a treatment for ALS [25, 26], (for more detail see next section).

The present review seeks to explore progress towards the implementation of precision medicine principles in drug development for the treatment of ALS. Critically, this review will address advances in our understanding of the genetic landscape of ALS and the importance of understanding population genetics and genetic testing when considering patient selection for clinical studies to ensure that the heterogeneity of ALS is duly addressed. Additionally, we discuss the advances in long-read whole-genome sequencing technology and how this can improve genetic discoveries including novel pathogenic variant and transcript detection, with examples from other diseases. We also discuss the recent advances in omics-data for understanding ALS patient sub-groups, propose a patient profiling workflow, and summarise key ethical considerations that are critical for equitable therapeutic development for ALS patients.

## Precision medicine for ALS: are we there yet?

*Precision medicine is defined as giving the right treatment to the right patients at the right time. *Over the past 20 years, there has been considerable progress in implementing precision medicine treatments, with the field of oncology setting the gold standard for this practice. As a result of advances in multi-omics technology and an emphasis on understanding the molecular landscape that characterises different tumours [27], it is now widely recognised that understanding the heterogeneity of cancer is paramount for the effective treatment of patients. For example, the advances in precision medicine approaches have now led to exponential approval of drugs by the FDA that are tailored to the specific mutational profile of lung cancer tumours, with 18 targeted therapies approved in the last 4 years [28]. As such, precision oncology has now evolved and replaced the conventional one-size-fits-all approach. Importantly, the foundations of increasing knowledge in molecular biomarker profiling will continue to aid in the development of new targeted treatments with the overarching goals that can be applied to any disease indication, including maximising clinical efficacy, minimising safety concerns, and reducing the associated economic burdens of disease management [29]. While precision medicine has been implemented with great success in the field of oncology, it is now becoming increasingly clear that this is the direction that is required for the treatment of complex neurodegenerative diseases, such as ALS.

Qalsody is the first ALS therapy that is targeted to a genetically defined sub-population of patients (1–2% of patients with *SOD1* variants) that has been approved based on the surrogate efficacy biomarker (NFL). Importantly, Biogen have initiated the ATLAS phase III study (NCT04856982) to evaluate the ability of Qalsody to delay the clinical manifestation of disease in presymptomatic carriers of *SOD1* variants [30]. The study uses biomarker evidence (rises in plasma NFL) to determine the predicted time of pheno-conversion [31, 32] for individuals with highly penetrant and fast progressing *SOD1* variants. The characterisation of the transition to prodromal disease will be critical not only for early intervention but because it is well known that there is considerable variability in both the age of disease onset and disease progression, even for highly penetrant variants in the *SOD1* gene [33]. Additionally, an important consideration will be the development of additional biomarkers to identify the 7% of patients that do not tolerate Qalsody to avoid the stimulation of known inflammatory reactions (myelitis, pleocytosis, increased immunoglobulin synthesis) [17, 34], particularly since neuroinflammation is a known contributor to ALS [35, 36], and stimulation of this additional trigger could have the potential to accelerate disease progression in these patients [37]. As such, one must consider the need for the development of an alternative SOD1 suppression therapy for the patients that do not tolerate Qalsody. Nevertheless, the ATLAS study will provide much needed insight on the efficacy of early intervention for treatment targeting genetic subgroups of ALS patients and will be the first of its kind within the field.

Aldesleukin is a recombinant human IL-2 that was previously approved by the FDA under the brand name Proleukin for the treatment of metastatic melanoma and renal cell carcinoma [18]. It was subsequently repurposed for ALS based on the known role of neuroinflammation at all stages of disease, particularly the correlation between neuroinflammation and ALS disease severity and progression [37, 38]. Low dose IL-2 has been shown to enhance regulatory T cell (T_reg)_ function, which is critical to control inflammatory mechanisms that contribute to neuronal injury [39, 40]. The IMODALS phase IIa trial initially investigated low dose IL-2 in 36 patients with ALS. Importantly, this trial was designed using an embedded experimental approach with an emphasis on incorporating biomarkers for target engagement and disease activity early in development (consensus guidelines [41]). The initial phase IIa trial met its primary end point demonstrating a dose dependent increase in T_reg_ cell numbers compared to the placebo group [25]. Of note, during this trial peripheral blood mononuclear cells (PBMCs) were collected at multiple time points for the analysis of transcriptome profiles to inform the characteristic expression changes of the patients responding to treatment. In conjunction, in vitro assays were conducted to assess the suppressive function of T_reg_ cells at different time points, which has been previously shown to indicate clinical status [38]. Using T_reg_ cell count post-treatment as the primary outcome measure, participants were classified into low, moderate and high responder groups. Analysis of the baseline transcriptome profiles of these different responder groups highlighted that expression differences were present at baseline and that patients that were least responsive to treatment had a more inflammatory profile at baseline. Treatment with low dose IL-2 resulted in a reduction in pro-inflammatory transcriptome profiles in both the high and low responder groups. Additionally, the identification of two genes (*TLR9* and *CD27*) that correlated pre-treatment expression with magnitude of drug response allowed the development of a target engagement two-biomarker based regression model to predict the primary outcome measure (T_reg_ cell increases post low dose IL-2 treatment) [42].

The recently completed Phase IIb MICROCALS trial further investigated the safety and efficacy of low dose IL-2 as an add on therapy to Riluzole, assessed over an 18-month period in people with ALS [26]. The trial was designed to include prespecified biomarkers including, T_reg_ count as a measure of target engagement, CSF levels of CCL2 as a measure of microglial activation and phosphorylated NFH (pNFH) as a measure for neuronal damage [26]. The trial did not meet its primary unadjusted endpoint showing a non-significant 19% decrease in risk of death following treatment. However, planned secondary analyses of the primary endpoint adjusted for significant prognostic covariates, revealed a significant interaction with pNFH levels. Stratification of patients based on CSF pNFH levels measured at the time of random allocation showed that IL-2 treatment was significantly associated with 48% decrease in risk of death in the 70% of the population that had low pNFH levels (750–3700 pg/mL), whist no survival effect was seen in patients with high pNFH levels (> 3700 pg/mL) [26]. This finding was further supported by significant changes in ALSFRS-R slope according to pNFH stratification. Further, this trial demonstrated target engagement by reporting a significant increase in T_reg_ count and decreased plasma CCL2 indicating a less inflammatory peripheral environment [26]. Interestingly, no difference was seen in CSF pNFH levels from random allocation to final treatment, however, the authors suggest this may be due to numerous reasons including, the turnover and clearance of CSF pNFH, the timing of CSF sample collection or the dosing regimen tested in the study. This trial emphasises the importance of including multiple prognostic biomarkers in the trial analyses since it can be impractical to stratify trial arms based on all relevant parameters in the initial instance [26]. The use of pNFH level as a stratification tool to address ALS heterogeneity will require further investigation and will provide a targeted recruitment strategy for future trials investigating the treatment effect of low dose IL-2 in ALS.

Lithium carbonate is an approved drug that is commonly used to treat psychiatric illness and has been shown to influence many pathways that are relevant in ALS, including the sprouting of neurons in the corticospinal tract, promotion of synaptogenesis and autophagy [43]. As such, lithium carbonate was previously examined as a treatment for ALS but was deemed to show no survival benefit in the general ALS population. However, in 2017, a meta-analysis of three lithium carbonate clinical trials showed that contrary to the reported negative outcomes, patients with the *UNC13A* (C/C) ALS risk genotype had responded, showing an improvement in survival [44]. This exploratory finding will now be validated in the Phase III MAGNET trial (NCT06008249) examining the potential of lithium carbonate to prolong the time to death or respiratory insufficiency in patients with ALS that are homozygous for the C allele [45].

The above clinical programs illustrate the importance of incorporating genetic information (i.e., genetically defined subgroups), the early inclusion of biomarkers and defined precision medicine strategies to identify and enrich for potential responders during clinical trial. Additionally, with the emergence of gene targeted therapies for ALS, it will be critical to incorporate these principles early in pre-clinical development to enable the greatest chance of success and generate the relevant data to understand how treatments may be expanded beyond the initial target population (patients with pathogenic variants) into additional sub-groups of sporadic ALS patients that are governed by similar disease mechanisms or molecular profiles.

## What we know about the complex genetic architecture of ALS

It is well known that ALS is a heterogeneous disorder with patients exhibiting variable clinical features including site of disease onset, age at disease onset, cognitive and behavioural changes in some instances, and variable survival duration [46]. With heritability estimates ranging between 37–66% depending on the parent–child dyads [47] or twin study [48], this phenotypic heterogeneity observed in ALS is underpinned by the interplay between the complex underlying genetic architecture consisting of rare pathogenic variants [49, 50], structural variants [51] and other changes in the non-coding genome, epigenetics [52] and the contribution of environmental/lifestyle factors [1] (Table 2).

**Table 2:** Glossary

Pathogenic variants: Historically referred to as ‘mutations’ but now there is a preferred shift within the field to refer to these genetic changes as pathogenic variants.
Repeat expansions: Genetic structural variation that involves repeat motifs that can occur hundreds or thousands of times in the genome and typically result in disease.
Genome Wide Association studies (GWAS): Studies which test millions of genetic variants across the genome and look at the association of genotypes with a trait of interest.
Polygenic risk score: Polygenic risk scores are calculated during GWAS and look at the weighted effect size of millions of single base pair changes in the genome and provide a value that describes the genetic liability for a trait of interest (ie, ALS).
Rare burden variants: Rare burden variants occur at a minor allele frequency of <1% in the population. Typical GWAS studies filter variants with a cut off minor allele frequency of between 1-5% depending on the study.
Structural variants: Typically considered >50bp encompassing different classes of genomic alterations including insertions, deletions, short tandem repeats, duplications, rearrangements, inversions and copy number variations.
Short structural variant: Structural variants that are <50bp in size.
Variant burden: Variant burden may refer to different types of genetic variation across a gene or genes that govern specific molecular pathways are increased in cases compared to controls.
Gene penetrance: The penetrance rate refers to the probability that an individual carrying the risk genotype will develop clinically manifest disease. Some variants have high penetrance meaning most individuals will develop disease, whilst others are low or incomplete penetrance meaning few individuals will develop the disease.
Disease modifiers: Genetic variation that may not contribute to the risk of developing the condition but that can influence the phenotype (ie, progression) of the disease.
Phenocopy: A disease that does not carry the relevant genotype but presents a similar phenotype or trait.
De novo variant: Variants that are not inherited from either parent or appear for the first time in a family.
Founder effect: Reduced genetic diversity that results when a newly established population is descendent from a small number of common ancestors.

### ALS classification

**Fig. 1:**
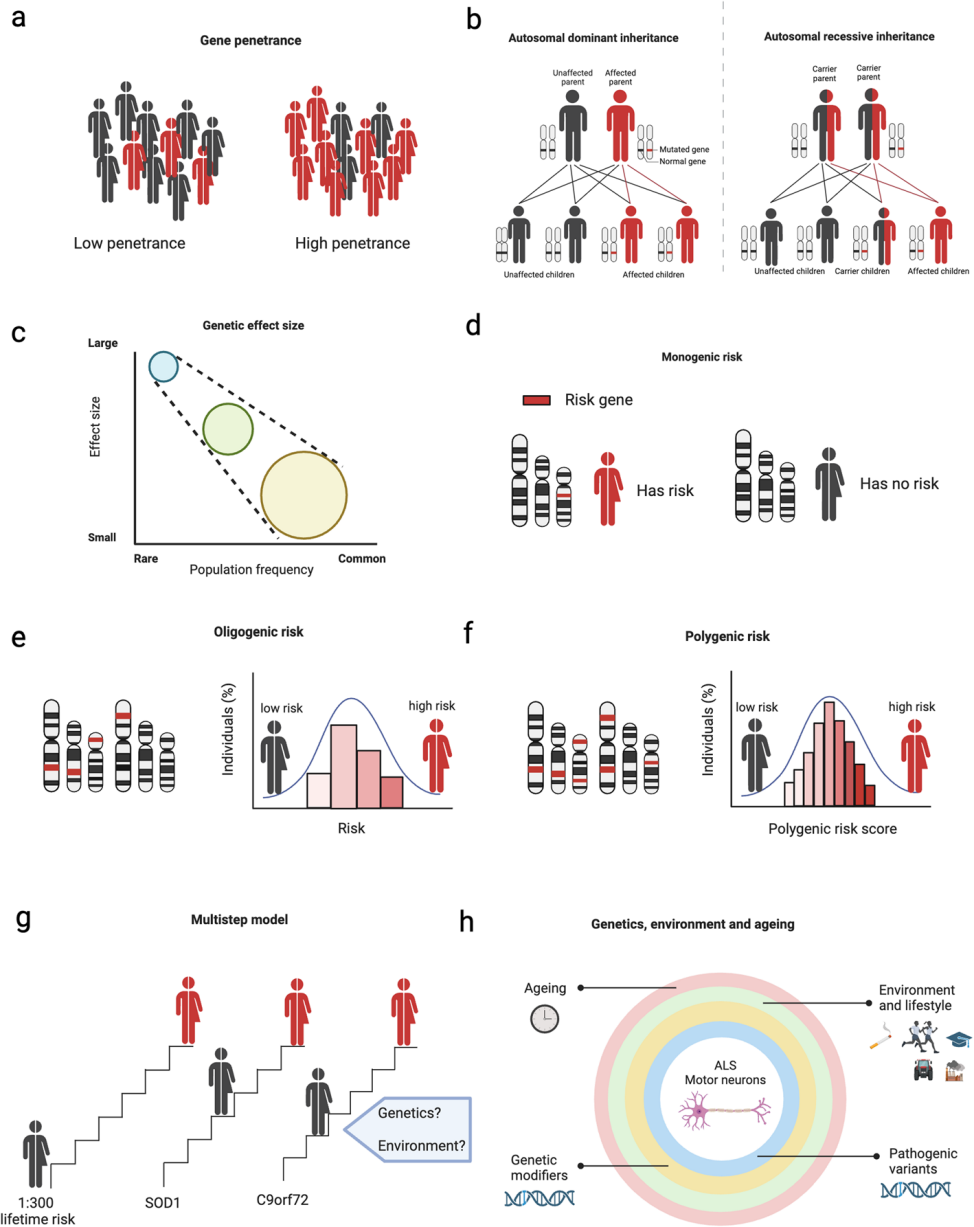
The genetic landscape of ALS. This figure has been created in BioRender and has been adapted from Dharmadasa, Scaber [14], Goutman, Hardiman [1] and Akçimen, Lopez [58]. **A** Genes involved in the pathogenesis of ALS are not fully penetrant meaning not all individuals carrying the risk gene/variant (all individuals) will manifest clinical disease (red), emphasising the complex genetic architecture of ALS. **B** ALS variants are typically inherited in a dominant or recessive fashion. Dominant inheritance requires only one inherited variant to result in affected offspring. Recessive inheritance requires both parents to carry one recessive variant for affected offspring. This example depicts heterozygous carrier parents whereby offspring that inherit both copies of the recessive variant will manifest disease. **C** The effect size of genetic variants relative to population frequency. Rare variants (blue circle) have a low frequency and large effect size, moderate frequency variants (green circle) typically have a moderate effect size and common variants (yellow circle) typically have a small effect size and contribute to polygenic risk. Circle size represents raw count of variants (**D**) Monogenic disease is characterised by the inheritance of variants in a single gene (**E**) Oligogenic disease is characterised by the inheritance of variants in multiple genes (**F**) Polygenic disease is characterised by the inheritance of variants in many genes that can cause an increase in the risk of disease (**G**) The multistep model of ALS depicting six molecular steps required to result in disease. Individuals with pathogenic variants (i.e. *SOD1* and *C9orf72*) require fewer steps to manifest disease. **H** An integrated model of ALS highlighting the overlapping contribution of genetics (pathogenic variants and disease modifiers, the environment and ageing)

Conventionally ALS is classified as either familial (prior family history) or sporadic (no prior family history). This historical classification system is becoming increasingly redundant since several factors can influence this classification, including ascertainment bias, family size, definition of relevant family history (ie, ALS vs ALS/FTD), early death of parents, estranged or undisclosed family diagnosis and incomplete penetrance [14, 53, 54]. Approximately 10–15% of ALS cases are considered familial with Mendelian inheritance [1, 46], which is often complicated by incomplete penetrance within kindreds (not every person with the genotype developing the disease Fig. 1A), while the remaining 85% of cases are considered sporadic [1, 46]. Although these classifications are still commonly used, with the emergence of gene targeted treatment for ALS, we anticipate that genetic testing will become a standard practice for all ALS patients and classification will shift to focus on variant status (known vs unknown) [1, 46, 55–57].

### ALS genes

Over 50 genes have now been associated with ALS (Table 3), with variants in *C9orf72, SOD1*, *FUS* and *TARDBP* being most common cause of ALS, while other disease-causing variants are relatively uncommon and represent < 1% of cases [1, 18, 58]. For most apparent sporadic cases, a clear genetic basis has not yet been identified, demonstrating disease complexity and heterogeneity. Only approximately 15–20% of sporadic cases can be explained by the currently known pathogenic variants in ALS [58, 129]. Since 2018, whole-exome and short-read whole-genome sequencing studies have enabled the discovery of 5 new ALS genes including *KIF5A*, *ERLIN1*, *DNAJC7*, *HTT* and *SPTLC1* (recently reviewed [58]). While these discoveries are promising, the confirmation of these being definitive ALS genes is still in process as indicated by the current ClinGen [130] (expert review panel) status for each gene indicated in Table 3.

**Table 3:**
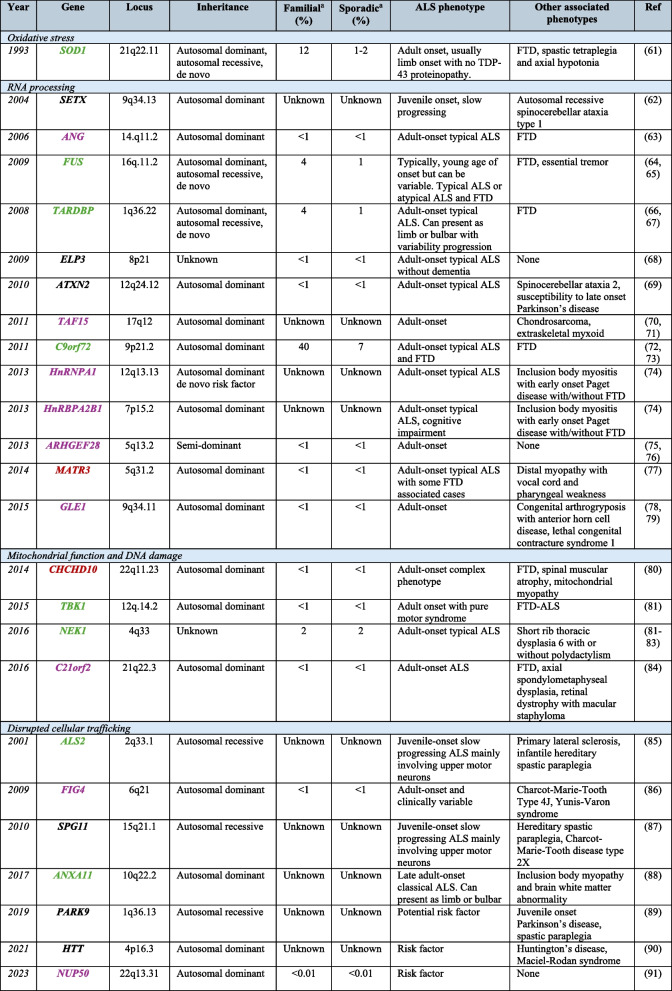

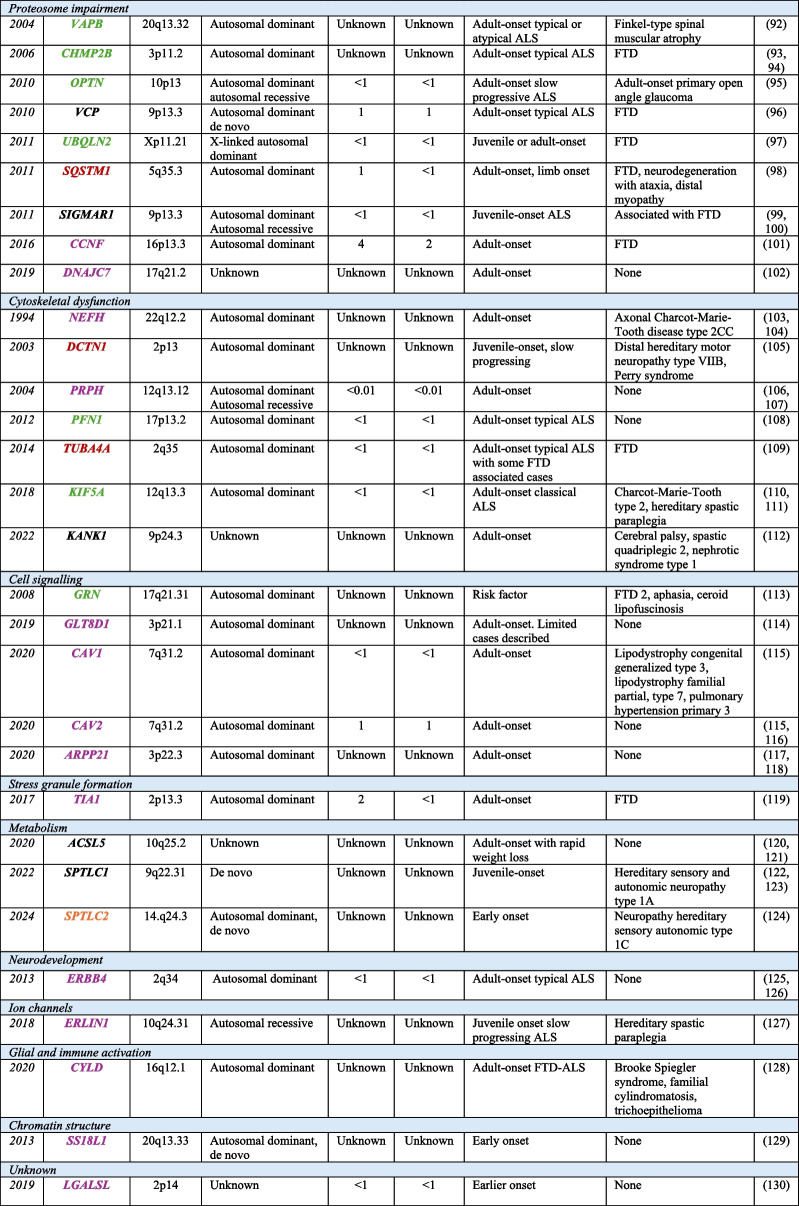
ALS genes [59–72, 72–79, 79–128]

Genes have been categorised by their primary disease-associated mechanism and listed chronologically based on the year of their discovery. ^a^Percentage of ALS cases explained by pathogenic variants in the corresponding gene. ^b^Phenotypes associated with the genes, taken from the Online Mendelian Inheritance Man database. Genes symbols have been colour coded according to the current ClinGen weight of evidence for ALS (Green = definitive, Orange = strong, Red = Moderate, Purple = limited, Black = curations not yet published). Genes that have been disputed or refuted are not included in this list

### Types of variation in ALS

While it is known that many ALS genes exhibit single point pathogenic variants with clear inheritance patterns (Fig. 1B), such as those in the genes *SOD1*, *FUS* and *TARDBP*, the importance of the non-coding genome and the contribution of structural variants is an emerging area in ALS research. The most well-known intronic variation in ALS is the repeat expansion in *C9orf72*, which accounts for the highest percentage of ALS cases in European populations. Similarly, expansions in *ATXN2* have also been associated with ALS [67, 131]. With the improvement in bioinformatic tools to size these repeats, this has paved the way for the identification of additional repeat expansions in *ATXN1* [132], *NIPA1* [133] and *HTT* [134] as risk factors for ALS. Similarly, these advances have also led to the identification of other classes of structural variation including an inversion in *VCP* and insertion in *ERBB4* that have been associated with ALS [123].

Variations in the non-coding genome may account for a signification portion of the missing heritability of ALS [51]. For instance, it is known that the untranslated regions of genes contain many essential regulatory elements that can influence transcript stability and exert post-transcriptional control, directing both cellular localisation and rate of protein synthesis [135]. The use of next generation sequencing has enabled the identification of an increased burden of rare variants in the untranslated regions (UTR) of many ALS genes including *SOD1*, *TARDBP, FUS*, *OPTN*, *VCP* and *UBQLN2* [50]. Additionally, variation in non-coding regions may also have domain specific effects, reinforcing the complex genetic architecture in some ALS genes. Recently a study employed both a meta-analysis of rare variants (minor allele frequency < 1%) previously reported in *NEFH*, as well as, exploring both rare and ultra rare variants (single nucleotide variants, SNVs and insertion deletions, Indels) present in *NEFH* in the large Project MinE data set [136]. Both rare missense variants in the section of the gene coding for the tail domain and intronic variants in the section coding for the rod domain were associated with ALS risk in the meta-analysis and Project MinE datasets. However, it was noted that the complexity of the *NEFH* domain architecture provided preliminary evidence that domain specific variation may be protective (KEP region [136–138]) diluting disease associations with variants in the tail domain (KSP region) [136], although further studies are needed to further elucidate this.

Further to influencing disease risk, non-coding intronic variants can also promote the inclusion of pseudo exons or impact the recognition of splice sites motifs that result in the aberrant splicing of transcripts. Aberrant splicing from intronic variants has been described for the ALS genes *SOD1* [139, 140] and *KIF5A* [109, 141]. Similarly, the inclusion of intronic sequence in the form on non-conserved cryptic exons is a known pathological feature of ALS [142], resulting from the loss of activity from RNA binding protein TDP-43. Inclusion of cryptic exons has been identified in many genes, with *STMN2* [143, 144] and *UNC13A* [145] being of relevance in ALS. Genome wide association studies (GWAS) have consistently replicated *UNC13A* as an ALS risk gene [82, 108, 146–148], with additional studies demonstrating a role in disease modification [149, 150]. Importantly, studies have shown that intronic variation near the cryptic exon sequence can create a risk haplotype that promotes its inclusion during states of TDP-43 depletion [145, 151]. Additionally, analysis of short structural variants in linkage disequilibrium with the top hit GWAS *UNC13A* loci (rs12608932 and rs12973192) suggest that a CATC repeat within the same intron (intron 20–21) makes *UNC13A* more vulnerable to cryptic exon inclusion and has been suggested as the main driver of disease risk [145]. This highlights the critical role of the non-coding genome in contributing another layer of complexity and in some cases enhancing pathogenic mechanisms in ALS.

In few sporadic ALS cases, de novo or spontaneous variants have also been described in *SOD1* [152], *FUS* [153], *VCP* [154] and recently *SPTLC* [120, 121]. Increased implementation of long-read whole-genome sequencing (see section on long-read sequencing) may improve the detection of types of genetic variation that are inaccessible with next-generation short-read sequencing (e.g., extended & complex repeats, inversions, and other forms of structural variation), and variants in complex regions of the genome (e.g., segmental duplications, repeat regions), leading to identification of novel ALS genes in the future. This may increase the gene/variation discovery related to 85% of sporadic ALS where no genetic variation is currently known.

### Pathogenicity of variants

Importantly, genetic variants associated with ALS can vary in their pathogenicity, with some being highly penetrant and others only influencing the risk of developing the disease over a person’s lifetime. As such, genetic variation occurs on a continuum of effect size and relative variant frequency [14] (Fig. 1C). Typically, rare variants will have a large effect size (ie, pathogenic variant causing monogenic disease, Fig. 1D), un-common variants will have a moderate effect size and may occur in a few genes to increase disease risk (oligogenic model, Fig. 1E), while common variants will have a low or negligible effect size and will influence disease risk through the sum effect sizes for many variants (polygenic risk score) across many genes (polygenic model, Fig. 1F) [1, 46].

### Variant classification

Variants are typically classified in the following categories, (1) pathogenic, (2) likely pathogenic, (3) variants of unknown significance, (4) likely benign and (5) benign. These categories provide guidelines for distinguishing the pathogenicity of variants and are based on the American College of Medical Genetics and Genomics Standards and Guidelines [55]. Assignment of individual variants to these classifications are based on numerous lines of evidence, including population frequencies, case control studies, clinical reports, co-segregation of the variant and disease within a family. Where a definitive disease mechanism is not available, in vitro cells models using patient derived material or animal models can provide evidence to support pathogenicity. If the various lines of evidence for a particular variant are conflicting or there is not enough evidence to assign it to any of the specified categories, then it defaults to uncertain significance. With the increased discovery power of long-read whole-genome sequencing, there will be a critical need to develop tools to prioritise newly identified variants of unknown significance (VUS), as previously inaccessible regions of the genome can now be characterised (see section on long-read sequencing). Undoubtedly, as genetic screening increases for ALS patients, it will increase the identification of both novel and rare variants that will require experimental validation and may inform the development of personalised treatments.

### Models of ALS (Gene-time-environment)

The multistep model of ALS suggests a series of sequential steps that drive the path towards disease, occurring from both genetic and environmental insults over time [155]. In European and Asian populations, the multistep model suggests up to six steps required to manifest disease (Fig. 1G), with fewer steps required in patients that harbour pathogenic variants that are highly penetrant (*C9orf72*, *SOD1*, *TARDBP*) [156, 157]. Additionally, the influence of sex, psychiatric illness (indicating overlapping genetic risk) and cardiovascular disease (indicating environment/lifestyle factors) may also contribute [158]. What remains unclear in this model is the precise timing of when a step has occurred [1], and additional work is required to further elucidate this. A recently proposed model by Akçimen, Lopez [58] represented this process as a series of concentric rings where motor neurone health lies within the centre (Fig. 1H). Each concentric ring represents interconnected factors that operate collectively to contribute to disease. Within this model, the contributing factors include pathogenic variants (formerly mutations) as the inner most ring followed by genetic modifiers, environmental and lifestyle factors and ageing. This model has been proposed to represent the synergistic nature of each contributor to disease and the interconnected forces that can lead to neurodegeneration [58]. While different models have been proposed to describe the occurrence of ALS, there is consensus on the factors involved in this process.

## Population genetics of ALS

It is well known that diversity occurs within the human genome, with both unique patterns and frequencies of genetic variation occurring among people with different ancestries. Unsurprisingly, the incidence of ALS also differs among populations with African, Asian and Hispanic ancestries having a lower incidence rate than European populations [159, 160]. It is currently unknown, whether these geographical differences are related to differences in epidemiological study design, environmental factors present in these distinct populations, differences in the exposome of individuals [161] or simply the result of the varying genetic architecture of ALS in different populations [162]. For instance, the pathogenic repeat expansion *C9orf72* is frequent in European populations but is rare in Asian populations (Table 4). Alternatively, pathogenic variants in *SOD1* and *FUS* are much more common in Japanese and Korean patients. The founder effect has not only led to the increased frequency of *C9orf72* in European populations but has also led to a the Ala282Thr variant in *TARDBP* being the most common cause of ALS in the island population of Sardinia, accounting for one third of ALS cases. Similarly, the Asp91Ala variant in *SOD1* is the most common pathogenic variants in the Scandinavian population and the Phe56Ser variant in *VAPB* accounts for 44% of inherited ALS in Portuguese-Brazilian populations.

**Table 4:** Frequency of pathogenic variants in the most common ALS genes across populations

	Europe		Scandinavia		Middle east (Iran)		USA		Canada		Australia	
	fALS	sALS	fALS	sALS	fALS	sALS	fALS	sALS	fALS	sALS	fALS	sALS
*C9orf72*	33.7%	5.1%	25.0%	4.5%	5.9%	1.60%	36.1%	5.9%	27.4%	3.6%	40.6%	2.9%
*SOD1*	14.8%	1.2%	28.1%	0.4%	35.3%	3.30%	19.4%	0.7%	17.0%	1.9%	13.7%	0.6%
*FUS*	2.8%	0.3%	0%	0%	-	-	3%	-	0.7%	-	2.4%	1.1%
*TARDBP*	4.2%	0.8%	0%	0%	-	-	2.8%	-	-	-	1.9%	0.8%
	China		Japan		India		Taiwan		Korea		Asia pooled	
	fALS	sALS	fALS	sALS	fALS	sALS	fALS	sALS	fALS	sALS	fALS	sALS
*C9orf72*	3.5%	< 1%	2.2%	2.2%	10.7%	2.8%	16.7%	1.5%	0%	0%	2.3%	0.3%
*SOD1*	25.6%	1.60%	32.0%	0.5%	9.1%	1.3%	26.7%	3.1%	54.8%	1.2%	30.0%	1.5%
*FUS*	5.8%	1.30%	8–11.0%	0.5%	0%	0%	6.7%	1.5%	5.4%	1.6%	6.4%	0.9%
*TARDBP*	5.8%	< 1%	2–3.5%	0.25%	-	1.3%	23.3%	-	0.3%	0%	1.5%	0.2%

The frequency of pathogenic variants in the most common ALS genes. References for each populations are as follows Europe (Belgium, France, Germany, Greece, Italy, Netherlands, Russia, Slovenia, Spain, Switzerland, Turkey, UK) [22, 162], Scandinavia (Finland and Sweden) [163], Middle east (Iran) [164], USA [162], Canada [162], Australia [165, 166], China [167, 168], Japan [162, 169, 170], India [162, 171], Taiwan [172], Korea [162, 173]

With varying ALS gene involvement across different geographical and ethnic populations, there is a clear need for the development of precision therapies that are relevant to each distinct patient population. In parallel, drug development must navigate differing jurisdiction-specific regulatory requirements, which can influence the type and quantity of evidence needed for approval, even when the targeted patient population is the same. These considerations will inevitably impact patient recruitment strategies for clinical trials.

## Importance of genetic testing for ALS patients

Genetic testing practices can differ between countries, with no formal consensus among specialists. While many clinics do offer genetic testing, this was historically reserved for patients < 40 years with a clear family history. This historical approach was complicated by the number of factors that can influence the classification of a positive family history (discussed previously) and the additional complexity of incomplete penetrance for some ALS linked variants [14]. As such, genetic testing based on these prior ALS classifications could miss clinically actionable results for up to 17% apparent sporadic cases [56, 174]. Importantly, genotyping of ALS patients regardless of their conventional ALS classification (familial vs sporadic), demonstrated that 21% of patients harboured a pathogenic variant, with 15% meeting the inclusion criteria for ALS gene therapies currently in clinical trial [56]. Importantly, this led to the revision of the UK genetic testing guidelines in April 2023.

Another important consideration that may influence the yield of clinically actionable results is the variation in the genes included in testing panels. In an assessment of 14 clinical panels, only 50% offered the option of including the *C9orf72* repeat expansion (the most common cause of ALS) [175]. This emphasises the potential for reduced diagnostic yields and missed diagnoses for patients, depending on the genetic panel that is used. With the growing number of ALS related genes, this also poses a significant challenge relating to the identification of variants of unknown significance (VUS), contributions of polygenic risk and the clinical interpretation and management of this information during genetic counselling. Moving forward, genetic testing practices will require an optimal approach to navigate these challenges.

### Overlapping neurodegenerative conditions and pleiotropy in ALS

The considerable overlap between ALS and other neurodegenerative diseases further supports the need for early genetic testing in all patients. This is of particular importance since several degenerative conditions are known to mimic ALS symptom presentation, including multifocal motor neuropathy, axonal motor predominant chronic inflammatory demyelinating polyneuropathy, inclusion body myositis and spinobulbar muscular atrophy [176]. Further considerations that can complicate the diagnosis of ALS have previously been reviewed [1].

While it is still necessary to diagnose patients based on clinical presentation, genetic testing can aid to predict the relative family risk or the cause of disease in some individuals [1]. However, ALS risk genes can also contribute to other phenocopy neurodegenerative diseases, which can further delay diagnosis of ALS. One example of this is *C9orf72,* which is the most common genetic cause of ALS, but can also result in other movement disorders including Huntington’s disease [177] and Parkinsonism [178, 179]. Alternatively, patients with ALS can harbour *HTT* repeat expansions [134], which typically cause Huntington’s disease [180]. Similarly, expanded repeats in genes associated with ataxia (*ATXN1* [132] and *ATXN2* [67, 131]) and hereditary spastic paraplegia (*NIPA1* [133]) are also risk factors for ALS. Further complicating the phenotypic overlap that can occur between movement disorders is the occurrence of pleiotropy, whereby variants in one single gene can result in distinct neurological conditions. A well-known example of this is the gene *KIF5A,* where genetic variation in N terminal can result in hereditary spastic paraplegia and Charcot-Marie-Tooth type 2 while genetic variation in C terminal can cause ALS [181–183]. Another example is *SPTLC1* gene where pathogenic variants can result in either the development of ALS [120] or peripheral neuropathy [184].

There is no diagnostic test for ALS, therefore, the accurate characterisation of repeat expansions may assist clinicians during this process, particularly where repeat sizing is paramount to confirming pathogenicity or increased risk for a given condition [185]. Recently, the company PacBio has released a comprehensive genotyping panel (PureTarget [186]) which focuses on the characterisation of repeat expansions using HiFi long-read sequencing. This panel includes 20 repeat expansion loci for the most medically relevant genes, including spinocerebellar ataxias, Huntington’s disease, ALS, FTD, spinal bulbar muscular atrophy and more. Since this panel utilises long-read sequencing technology (discussed in next section), it enables the accurate sizing of both alleles (including large expansions) and providing accuracy down to a single base resolution (necessary for resolving repeat interruptions in imperfect repeats) with deep coverage and the ability to profile mosaicism within a given sample. Leveraging these technologies may assist clinicians in uncovering genetic causes of disease or genetic risk factors for cases that were previously considered sporadic. For instance, a recent study identified four patients with *ATXN2* intermediate expansions through retrospective genetic screening of bio-banked DNA samples and one patient through prospective testing, with none of the five patients having a reported family history of ALS [187], further emphasising the importance of genetic testing. While the PureTarget panel is currently available for research use only, use of this panel may help to further understand the complex genotype–phenotype relationships in neurodegenerative disorders, which will ultimately expedite the diagnosis of ALS in the future.

## Long-read sequencing will revolutionise our understanding of ALS

Short-read sequencing technologies have dominated the field over the last 10 years. While whole-genome sequencing studies have greatly improved with the implementation of PCR-free library preparation reducing the effects of amplification bias, the main limitation of this method is related to the size of individual read lengths (150–200 bp on Illumina platforms), leading to a skewed picture of genomes focused on SNVs, Indels and CNVs (copy number variations), but being challenged in resolving other genomic features. In recent years, long-read sequencing has demonstrated superior performance in sequencing genomes [188–190]. Designated “2022 Method of the Year” by the journal *Nature Methods* [191], long-read sequencing has matured, and the dramatically increased accuracy, ever-increasing throughput and access now allows new and advanced studies, even at scale. This has led to a steadily increasing amount of human genome and transcriptome studies being enabled [192]. Long-read sequencing technologies are currently available from Pacific Biosciences (PacBio) and Oxford Nanopore Technologies (ONT), with both PacBio and ONT technology platforms previously reviewed [193]. PacBio Hifi sequencing has the highest median accuracy of any sequencing platform (99.9%) compared with ONT (ranging between 87–98%), evidenced through recent benchmarking [188–190, 194–198]. For the purpose of this review, we will only discuss PacBio Hifi sequencing and its potential application to variant discovery.

HiFi sequencing was the enabling technology used to generate the complete sequence of a human genome, contributing an additional ~ 8% to the human genome sequence [199]. Subsequently, it was used to create the first human global [200] and population-specific [201–203] pan-genome references, adding hundreds of millions of bases that were not present in the human genome reference, thus highlighting the full extent of genetic variation across human populations, and covering all types of genetic variation – SNVs, Indels, different forms of structural variation (SV ie, insertions, deletions, inversions, tandem repeats, translocations), and complex regions. The long reads also allow separation and phasing of the two alleles, resulting in a fully resolved 6 Gb genome, rather than previously collapsed 3 Gb representations [204], and allowing for the resolution of multiple variants in *cis vs. trans*, critical for the study of recessive diseases.

Two examples of the dramatically improved resolution of human genomic features through HiFi long-read sequencing are (1) Segmental duplications – highly homologous, relatively recently duplicated regions (critical for brain evolution and often implicated in CNS diseases). Using HiFi sequencing, the human pangenome reference consortium extended variant calling into 120 Mb of additional segmental duplication sequence per genome, identifying ~ 2 million nonredundant SNVs in a gene-rich portion of the genome previously considered largely inaccessible [205]. Critically, they observed that SNVs are elevated 60% in segmental duplications compared to unique regions, i.e. these regions are in fact more variable than those studied traditionally with short-read sequencing. (2) Tandem repeats – the most common type of structural variation, encompassing the largest number of variable bases between humans, and expansions thereof responsible for many CNS diseases. Difficult to assess with short reads, HiFi sequencing has been used to generate the first comprehensive catalogs of tandem repeats in human genomes and across populations [206, 207], and appropriate tools developed to analyse and standardise both simple and complex tandem repeats.

Further, long-read sequencing machines (both PacBio and ONT) are able to use natural and untreated DNA, outputting 5mC (methylation) in addition to the four canonical bases as the standard sequence, allowing for linking epigenetic sequencing and DNA sequencing in a high-resolution view of function and genetic variation [208]. The application of long-read sequencing has potential in both rare and complex diseases because we know that 90% of susceptibility to common disease is encoded in the regulatory DNA rather than the coding DNA [209]. For the above reasons of improved variant calling accuracy, HiFi sequencing has been used increasingly for the identification of the underlying genetic causes of rare and inherited diseases [210–212], for the characterisation of biobank samples [213], for large-scale national programs [214], and for providing answers for CNS diseases (see Table 5 for examples using HiFi sequencing).

**Table 5:** Examples for genetic answers in CNS diseases unlocked through HiFi long-read sequencing

Huntington’s disease: Long somatic DNA-repeat expansion drives neurodegeneration in Huntington disease [215]. Huntington’s disease is a fatal neurodegenerative condition in which striatal projection neurons involved in regulating mood and movement begin to degenerate. One of the longest standing biological questions in Huntington’s disease is how the DNA repeat expansion (CAGn) in the huntingtin gene (*HTT*) can lead to neurodegeneration after decades of apparent latency. Long-read sequencing has enabled the precise measurement of these CAG repeat lengths in single cells and has linked repeat length to each cells’ respective gene expression. Handsaker, Kashin [215] demonstrate that somatic expansion from 40 to 150 CAGs have no effect on gene expression but once neurons cross a threshold of 150-500+ CAGs it results in profound gene expression changes, particularly in genes responsible for senescence and apoptosis. The authors conclude that the *HTT* CAG repeats undergo decades of ‘biologically quiet’ expansion and once the repeat passes a certain length threshold, it causes rapid and asynchronous degeneration of striatal neurons.
Parkinson’s disease: The diversity of *SNCA* transcripts in neurons, and its impact on antisense oligonucleotide therapeutics [216]. Parkinson disease (PD) is an incurable condition characterised by the progressive loss of dopaminergic neurons in the mid brain and aggregation of protein a-synuclein (encoded by *SNCA*). Using long-read sequencing, Evans, Gustavsson [216] showed that previously annotated *SNCA* transcripts only accounted for 5% of SNCA expression. Importantly, the majority of SNCA expression (75%) originated from transcripts with alternative 5’ and 3’ UTRs, with 10% of transcripts originating from open reading frames that were not previously annotated, that were subsequently detected as novel protein isoforms in human postmortem brain. The authors emphasised that defining of the 3’UTR enabled the design of ASOs that could target the majority of *SNCA* transcripts leading to the effective reversal of PD pathology, including protein aggregation, mitochondrial dysfunction and toxicity in vitro models. This example highlights the importance of resolving the transcriptional landscape of proteins prone to aggregation in neurodegenerative disease and how this can inform the development of more targeted and efficacious precision ASO therapies.
Parkinson’s diseases: Accurate long-read sequencing identified *GBA1* as major risk factor in the Luxembourgish Parkinson’s study [217]. Heterozygous variants in the *GBA1* gene have been increasingly recognised as a risk factor for PD. One of the major challenges ofgenotyping variants in *GBA1* is due to a pseudogene which shares 96% homology to the coding region of GBA1. Pachchek, Landoulsi [217] used long read sequencing to characterise the full landscape of *GBA1*-related parkinsonism in Luxembourg and highlighted the high prevalence of *GBA1* variants as a major PD risk factor. This study demonstrates the important advancement of highly accurate *GBA1* variant calling using long-read sequencing, which was not previously possible using array-based or short-read sequencing methods. Moving forward, accurate variant genotyping will be essential for providing access to emerging therapies for *GBA1* carriers.
Schizophrenia/bipolar disorder: Characterisation of human-specific tandem repeat associated with Bipolar disorder and Schizophrenia [218]. Bipolar and schizophrenia are heritable diseases that have a genetic overlap with ALS. Genome-wide association studies have repeatedly linked risk of these psychiatric conditions to a 100kb interval in the third intron of the *CACNA1C* gene, however the causative mutation was not yet known. Song, Lowe [218] identified a human specific tandem repeat that was previously collapsed in the human reference genome. Leveraging long-read sequencing the authors were able to show that the 30-mer repeat region was polymorphic in both size and sequence, with variants of this repeat being associated with risk status and several flanking SNPs that have previously been identified in GWAS studies. This study emphasis that long-read sequencing can resolve complex and unstable repeat regions that were not previously sizeable and provide additional clarity to the genomic region of interest.
Batten disease: *CLN3* transcript complexity revealed by long-read RNA sequencing analysis [219]. Batten disease is a group of rare inherited diseases of which Juvenile CLN3 disease is the most prevalent type. The most common pathogenic variant in Juvenile CLN3 disease is the 1kb deletion which removes two internal coding exons (exons 7 and 8) in the *CLN3* gene. Zhang, Minnis [219] resolved the full range of *CLN3* transcripts in 99 healthy individuals. Leveraging long-read RNA sequencing the authors identified 172 *CLN3* transcripts, of which 147 were novel and 48 *CLN3* open reading frames, of which 26 were novel. Surprisingly, the major disease associated transcript (‘Δex7/8’) was also identified across 22 control samples. This study highlights the importance of studying both the canonical and non-canonical isoforms of critical transcripts and proteins, as well as the regulatory role of the UTRs to fully understand gene expression and regulation. This study provides critical insights that will be necessary for the future studying of patient samples, providing a reference of *CLN3* transcripts that will assist in the understanding of how the 1kb deletion affects *CLN3* transcription and contributes to disease pathogenesis.

Long-read sequencing has begun to be utilised in ALS research [220]. HiFi sequencing has been used in numerous studies, either by WGS or by amplification-free targeted sequencing, to resolve the hexanucleotide GGGGCC repeat in the *C9orf72* gene [221–223], demonstrating allele-resolved sequencing of the repeat lengths (even for repeats in the thousands), alongside with their single-molecule resolved length distributions and associated methylation status. It has also been used to determine changes to the gene locus after gene editing [224], and in an exciting recent study describing the reversal of *C9orf72* mutation-induced transcriptional dysregulation and pathology in cultured human neurons by allele-specific excision, it was used to size the repeat expansion, detect repeat expansion excision in C9-REx and detect methylation after editing across *C9orf72*-patient lines [225]. With the ability to detect methylation changes, the continued uptake of long-read sequencing will provide new and exciting developments regarding the emerging role of epigenetics in ALS [52, 226, 227].

ALS-associated SVs have also been explored, e.g. a human-specific 69 bp variable number tandem repeat (VNTR) associated with ALS in the last intron of *WDR7*, exhibiting striking variability in both copy number and nucleotide composition [185]. Greater repeat copy number was significantly enriched in three independent cohorts of individuals with sporadic ALS, and that some repeat segments are solely present or absent in certain geographic populations. This example highlights the new opportunities to study mechanisms of repeat expansions and a framework for evaluating the roles of VNTRs in ALS.

Other structural genomic elements have been studied with HiFi sequencing, e.g. improved resolution of human endogenous retroviruses (HERVs) for which long, accurate reads are critical to resolve the large (~ 7 kb) and repetitive retroviral sequences. The merits of PacBio sequencing in the resolution of per-locus HERV expression has been described [228]. Already demonstrated in HIV patients [229], accurate sequencing of HERVs will likely provide unique opportunities to better define the roles of these retrovirus-derived genomic regions in the pathophysiology of ALS, and to determine whether variants in these regions contribute to ALS [58], as well as connections to HIV-associated ALS [230].

Similarly in the field of RNA research, decades of short-read RNA-seq have focused on gene expression counting, at the expense of understudying the effects of extensive RNA transcript alternative splicing and other RNA processing. However, it has been shown that alternatively spliced isoforms from the same gene behave as if encoded by distinct genes rather than minor variants of each other [231], and there are numerous examples of two isoforms of the same gene having the opposite biological function [232–234]. Over the past few years, full-length long-read RNA sequencing has shed light on this critical biology, resulting in the renewed appreciation that transcript isoform expression, rather than gene expression, is a key driver of biology and diseases. For example, PacBio’s full-length Iso-seq method was first utilised to build comprehensive, isoform-resolved transcriptome references [235–237] highlighting the previously hidden complexity of the human transcriptome. It is now used increasingly to understand CNS diseases [238–240] (see Table 5 for additional examples). Information about novel transcript isoforms as well as the ability to accurately characterise variation in the UTR is critical for the effective design of ASOs to either suppress all transcripts or elicit isoform specific activities.

Recently, a workflow was demonstrated to generate four comprehensive, allele-resolved human ‘omes – the genome, 5mC epigenome, chromatin architecture, and full-length transcriptome – from a single HiFi sequencing run, enabling for the first time a truly integrated, synchronous acquisition of multi-omics data for a comprehensive understanding of the genomic, epigenomic and transcriptomic constitution of a biological sample, and their causal relationships and interdependencies of causing several disease phenotypes [241].

## Multi-omics for biomarker discovery and patient stratification

Multi-omic approaches include the detection and or quantification of multiple biological systems including genes (genomics), mRNA (transcriptomics), proteins (proteomics), epigenetic factors (epigenomics) and metabolites (metabolomics), providing a complex integration of different layers of biology that can provide a holistic picture of a complex disease. While the genomic landscape has been extensively surveyed providing a wealth of information of the genes/pathways involved in ALS [82, 148], recent studies are seeking to further understand distinct genomic and molecular signatures that may underpin the heterogeneity seen in ALS [110]. Here we will discuss the implications of four key papers and their contribution to understanding the genetic and molecular underpinning of ALS sub-groups.

One such study used unsupervised machine learning latent cluster analysis to identify distinct patient clusters based on patient phenotype information. Spargo, Marriott [242] conducted the largest study to date and demonstrated that ALS patients can be clustered into five distinct phenotypically defined subgroups (regardless of clinical diagnosis ie, PLS, PMA, ALS), with each subgroup displaying distinct genetic architectures. Interestingly, the *C9orf72* expansion was more frequent in cluster 1 and *SOD1* variants were overrepresented in cluster 2, with certain clusters also being associated with a higher polygenic risk score for Parkinson’s disease, demonstrating overlap between other neurodegenerative disease risk genes that may only be relevant in distinct sub-groups of patients [242]. In addition, cluster 1 (*C9orf72* cluster) and 2 (*SOD1* cluster) were also associated with differential biological mechanisms highlighted by RNAseq from the motor cortex of ALS patients, with enrichment analysis capturing different dysregulated gene sets within each cluster [242]. As such, evidence is now mounting that ALS phenotype may present differently according to the disruption of sub-group specific cellular processes [243], with studies reporting differences in disease progression according to common variants in inflammatory and antioxidant pathways [244, 245], and gene expression-based clusters [243, 246, 247]. One possible implication of this study is that there may be the ability to identify patients that have ‘SOD1 like’ features using baseline clinical data, identifying sALS patients that could be amenable to the SOD1 targeted therapy Qalsody or other SOD1 targeted therapies currently in pre-clinical development.

Building on previous molecular sub-grouping studies [244, 248–250], Marriott, Kabiljo [247] used unsupervised machine learning methods to investigate RNAseq data from motor cortex and blood from sALS patients. Three distinct molecular sub-groups were identified based on the clustering of gene expression profiles, including (1) synaptic and neuropeptide signalling, (2) oxidative stress and apoptosis, and (3) neuroinflammation, with each molecular phenotype being reinforced by cell compositions relevant to the pathways identified within each patient cluster [247]. Importantly, this study validated molecular sub-groups in multiple independent data sets, leveraging both post-mortem and pre-mortem samples to ensure that clustering based on gene expression profiles from end stage disease (motor cortex) is also relevant during the earlier phase of disease, when analysing pre-mortem tissue (blood) [247]. Additionally, the molecular sub-groups were also tested for discriminative power and were able to distinguish between cases and controls, indicating that these molecular phenotypes have diagnostic potential, however further studies are needed to fully understand the nuances of these molecular phenotypes throughout the course of disease [247]. While this study supports the notion that ALS is not a singular disease and patients fall into distinct molecular sub-groups based on ALS pathogenesis, what remains unclear is whether a patient stays within a particular molecular cluster throughout the course of their disease or if this can change as the disease progresses. Nevertheless, the strong assignment between blood and motor cortex demonstrated in this study will enable the changes in blood expression profiles to be tracked throughout a patients’ disease to answer this question. Finally, in a similar manner as above, the identification of distinct molecular subtypes linked to oxidate stress could aid in the selection and recruitment of sALS patients for clinical studies that may also be amenable to a SOD1 based therapy, broadening the treatable population beyond the current criteria of only patients harbouring pathogenic *SOD1* variants.

Similarly, omics data from the prefrontal cortex of ALS patients have recently been investigated to try and capture molecular sub-groups based on an earlier stage of disease (only affected by TDP-43 pathology in a later stage of disease) [246]. Four molecular sub-groups were identified with notable differences in gene expression and splicing patterns occurring between sexes [246]. These different clusters showed some overlap with molecular pathways described in previous studies [244, 247]; however, the divergence of some pathways may indicate capturing a different stage of the disease process. The post-mortem omics data was also compared to omics data from cortex samples from mouse models of *SOD1*, *C9orf72*, *TARDBP* and *FUS* ALS. Again, some overlap was identified between the mouse and human omics data, although critical aspects of the disease, such as cryptic splicing could not be investigated due to differences in mouse splicing machinery and the transcriptomics read coverage in the human dataset [246]. Although, mouse models will not be able to fully recapitulate the molecular sub-group seen in humans, this study demonstrates that the most appropriate mouse model can be selected for in vivo modelling depending on the mechanisms of the drug of interest and the molecular sub-groups of patients that are relevant [246].

Finally, Kumbier, Roth [251] investigated the use of phenotypic and molecular features of fibroblasts as a proxy for patient response to ASO treatment in *FUS*-ALS (pathogenic variants in *FUS* gene). The authors used machine learning classifiers trained from high content imaging and transcriptome profiles to distinguish *FUS*-ALS fibroblasts from healthy controls. Not only did *FUS*-ALS fibroblasts show distinct features associated with disease, cells that had higher clustering scores also corresponded to patients with an earlier age of disease onset (including pre-symptomatic patients) [251]. Patients that had higher image clustering scores demonstrated a stronger response when treated with a FUS targeted ASO and shifted in clustering profile towards that of the healthy control fibroblasts [251]. Similarly, sALS fibroblasts that had a clustering profile similar to *FUS* fibroblasts also showed a stronger response when treated with the *FUS ASO* compared to the sALS fibroblasts that did not sit near this cluster [251]. While this study is pilot in nature, it shows the potential for using easily accessible cells (skin cells) to rapidly screen ASOs candidates and predict potential responders, beyond that of mutation positive patients. While this study provides evidence for rapid drug screening in a high-throughput platform, there are still some questions remaining. The first being, it is currently unclear how the response in patient fibroblast will correlate to the drug response in a more relevant cell type (ie, patient derived motor neurons) and how this model correlates with actual response in the clinic. Secondly, while this approach may provide a rapid and cheaper drug screening model for some drug targets, this methodology would not be suitable for axonal based targets that are not readily expressed in fibroblasts.

The above studies demonstrate the recent advances in leveraging omics data and machine learning methodologies to better understand both phenotype and the underlying molecular heterogeneity between ALS patients. While this is greatly improving our understanding of ALS disease sub-groups, it will be critical to ensure the development of standardised workflows (see next section) to create large and comparable data sets, as we untangle the complexities of this disease in different populations.

## Patient profiling in ALS drug development

With the increase in affordability and uptake of long-read sequencing, the ability to detect novel genetic variants will improve tenfold, providing data that was not previously accessible by short-read sequencing technologies. To leverage this new genetic information, it will be paramount to have improved reference genomes which capture the broad spectrum of genetic variation across different ancestries, so that as we enter the era of precision medicine, no patients are left behind. Projects including the draft human pangenome reference [200], the pangenome reference of Chinese ethnicities [201], a draft Arab pangenome reference [203], and a Pacific Islanders pangenome [202], are now beginning to fill this gap. With the increasing depth of accessible genetic information, it will assist our understanding of why shared genetics can translate into different phenotypes in different people. Culminating in the improved detection of genetic variants, identification of genetic modifiers, and the intersection of polygenic risk and epigenetic factors, it will help to unravel the complexities of the incomplete penetrance of pathogenic variants in ALS.

Bioinformatics tools that enable deep variant hunting and prioritisation to help predict functional consequences of both genetic and epigenetic changes will be critical for the prioritisation of VUS in ALS. This will assist in selecting genetic candidates for experimental validation and the potential of identifying new targets for therapeutic development, specifically tailored to individual patients. Similarly, bioinformatic platforms have emerged to mine large datasets across transcriptomics and clinical information that will aid in the clustering of patients with distinct profiles. One such example is the Deep Integrated Genomics Analysis Platform (DiGAP™), developed by GenieUs Genomics [252], to provide a comprehensive genomics profiling system to stratify ALS patients. The DiGAP™ platform in being used in the upcoming Phase 2 clinical trial, ROAR-DiGAP (NCT06429059), which is aimed at advancing the use of precision medicine approaches in ALS research. Using DiGAP™ genomic profiling, patients will be stratified into four distinct groups according to their Pathway Mutation Burden in various biological categories relevant to ALS pathophysiology (neuroinflammation, oxidative stress, impaired autophagy and axonal transport and mitochondrial dysfunction). Each patient group will receive individualised treatment tailored to the pathway category. In addition to the initial patient stratification, the DiGAP™ platform will also be used to uncover potential genetic signatures that may also serve as genomic biomarkers for responders and non-responders to each treatment.

**Fig. 2:**
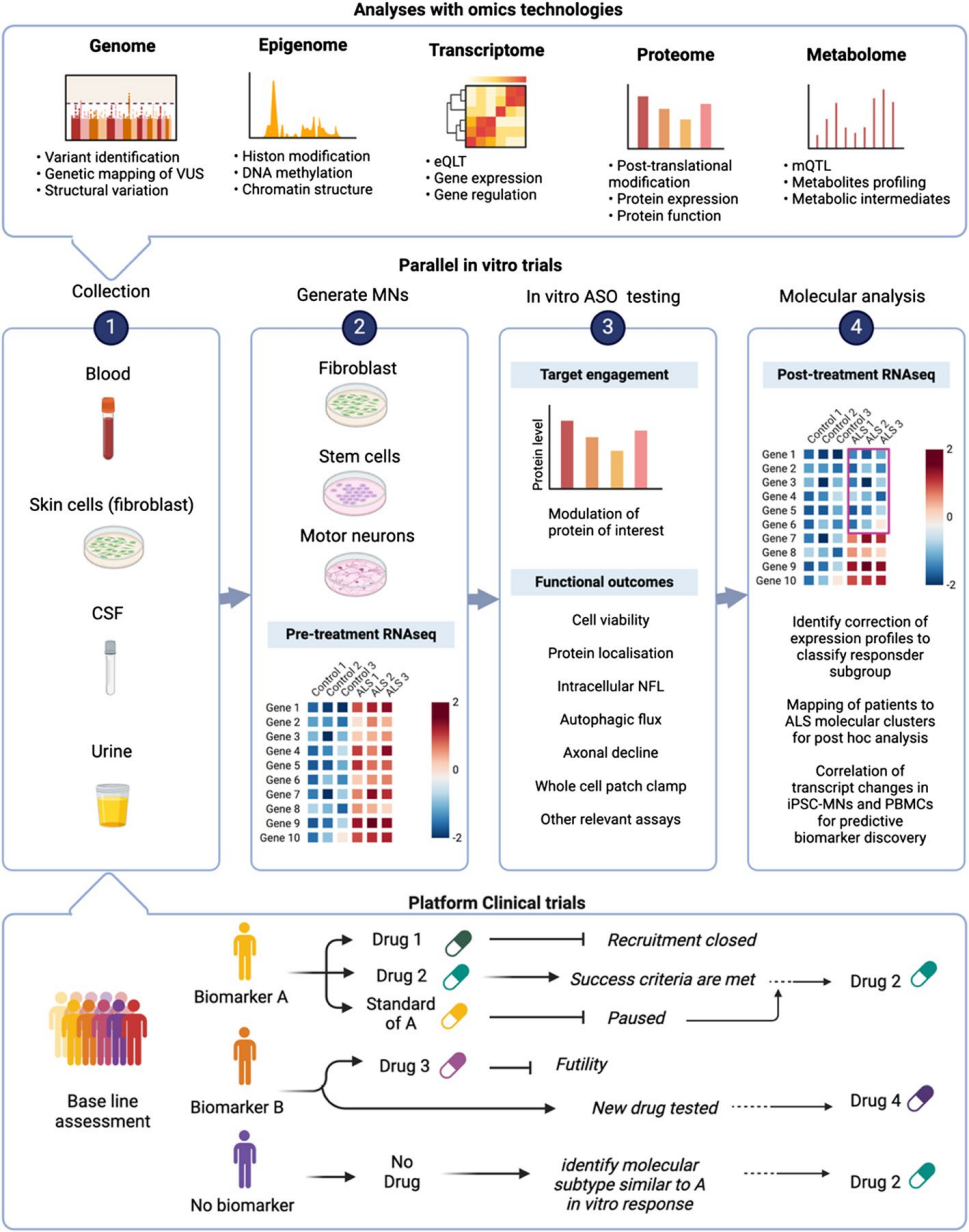
Precision medicine for ALS leveraging multi-omics, in vitro trials and platform clinical trials. This figure has been created in BioRender. Both top and bottom panels feed into Box 1, with biological samples being collected from patients at baseline (bottom box) and investigated using multi-omic approaches (top box) including, long-read whole-genome sequencing analysis, epigenome analysis, transcriptome analysis, proteome analysis and metabolome analysis. Box 2, Patient samples enable the generation of patient derived motor neurons (from stem cells or direct conversion from fibroblasts to induced neurons) and RNAseq conducted on motor neurons and other relevant biofluids (ie, blood) to provide baseline molecular information and allow in vitro testing of relevant gene modulating therapies. Box 3, Patient neurones should be tested with gene modulating therapies (ie, ASOs) to determine target engagement and provide indications of efficacy through various functional assays that indicate potential responders. Box 4, post-treatment RNAseq analysis of patient motor neurons will determine the correction of dysregulated gene expression pathways. Molecular analysis of biofluids post clinical treatment will also allow additional post-hoc testing to determine the shifting of previously described molecular subgroups of sALS patients. Molecular analysis should occur alongside early clinical trials as this will help in identifying predictive biomarkers through mapping the correlation of RNAseq changes in patient motor neurons post-treatment and the changes in RNAseq of patient bloods post-treatment in the clinic. These in vitro assessments should occur alongside clinical studies ensuring that patients are assigned to the most appropriate treatment as more gene modulating therapies enter clinical trials for ALS. This will also ensure the correct patient sub-groups receive the most personalised treatment and as data accumulates, this model would adapt so that patients can be recruited and enrolled according to their molecular subtype and/or PBMC predictive biomarker status ahead of time

While our understanding of the molecular sub-groups in ALS continues to improve, it will assist in the tailoring of treatments to both the genetic and molecular signature of patients. While we continue to unravel these complexities, it is our view that full omics data should be captured during in vitro testing of drugs in patient derived motor neurons (Fig. 2). This should be conducted alongside early clinical studies and will assist in identifying responder vs non-responder subgroups and will allow additional information to be captured during a patient’s enrolment into a clinical trial. Once the evidence continues to mount for targeted therapies based on ALS genetic/molecular subgroups, the suggested workflow will evolve so that patients are specifically recruited or triaged from other studies onto the most appropriate drug, for their genetic and molecular form of ALS.

## Ethical considerations

As we have noted, despite advances in understanding its pathology, treatment options for ALS remain limited and largely palliative. However, emerging tools in genetic assessment and intervention, such as those described herein, provide a promising opportunity to both deepen understanding of ALS, and translate this knowledge into transformative therapeutics. These developments are not merely scientific milestones but represent a profound ethical imperative for medicine [253, 254]. Given the genetically heterogeneous basis of ALS, advances in whole-genome long-read sequencing and genome-wide association studies are crucial to identifying key genetic variants that play a role in the pathogenesis and progression of the disease.

Ethical benevolence establishes that extant knowledge gaps be addressed in order to fortify the relative good(s) of improved diagnostic accuracy, identifying at-risk populations, and advancing precision-targeted personalised treatments [255]. Such comprehensive genetic assessments can reveal novel genetic patterns, complex gene-environment interactions, and disease mechanisms that heretofore have remained obscure. Expanding the foci, scope and availability of genetic assessments well aligns with the principle of justice. Simply put, absent a more granular understanding of ALS genetics, treatments will disproportionately benefit those whose genetic profiles have been well-studied, often populations of European ancestry. Broader research efforts, including diverse genomic studies such as those detailed in this paper, are ethically important – if not necessary—to ensure that emerging therapies are safe, effective and accessible for all ALS patients, regardless of their demographics.

The application of newfound genetic information to develop population specific treatments represents a transformative opportunity to shift ALS care from symptom management to addressing the patho-etiologic foundations of the disease. Technologies such as antisense oligonucleotides (ASOs), CRISPR-Cas9 gene editing, and gene therapy hold promise for mitigating or overcoming the effect of pathogenic variants. Yet, these innovations must be developed and applied in ways that are ethically sound. Genetic findings must be accurate, reproducible, and actionable. This necessitates stringent standards for genetic testing, including validation of new biomarkers such as those described, to reduce false-positive and/or false-negative results, and robust preclinical research – and prospective studies—to ensure that gene-targeting therapies are safe, and effective, minimising risks of off-target effects and unforeseen burdensome and/or harmful complications. Any and all pre-emptive and iterative cautions viably sustain nonmaleficence, fortifying the moral obligation to minimise harm in the development and application of genetic interventions.

It is important to note that genetic assessments and gene-based therapeutics for ALS entail numerous implications for patients and their families, which primarily obtain issues of autonomy, and informed consent. Ethico-legal respect for autonomy dictates that competent individuals have a right to exercise control over decisions related to their health, inclusive of genetic testing and the engagement of gene-based therapies. Informed consent is fundamental to respecting autonomy in such contexts, and patients must be provided clear, comprehensive information about the benefits, limits, burdens and risks of genetic testing. This includes the possibility of identifying incidental findings, such as risks for other genetic conditions, implications for family members with similar genotypes, and issues arising if and when treatments are not (yet) available for identified genetically induced pathologies. When treatment options are available, patients must be afforded opportunity to weigh the benefits and risks of experimental treatments, and to make decisions based on their values, goals, and preferences. Often, however, such burdens and risks may not be fully known/understood, given the novelty of emerging interventions, and we have argued that at very least, patients must be explicitly informed whether, and to what extent continuity of prospective research and clinical care of their individual condition will be made available [256].

Beyond apparent idiosyncratic ethical considerations, the development, articulation and provision of emerging assessment and therapeutic tools and techniques must be considered in more broadly systemic perspective [257]. As with new methods and targets of genetic assessment, emerging therapeutics should be available to those patients who require them. However, such a widely commutative form of just allocation may be difficult to execute absent an extant system of uniform provision of health care. Even then, the relatively high cost of developing and deploying genetic therapies presents significant macroeconomic, microeconomic and thus ethical challenges. Any applied construct of distributive justice must account for these factors, and this prompts query of how such state-of-the-science therapeutics can be made accessible to all who need them, rather than being limited to those who can afford them.

Addressing these disparities requires collaborative efforts between researchers, the commercial enterprise, healthcare providers, policymakers, and the economic sector. We have posited previously [258, 259], and re-iterate here that ethical strategies such as tiered pricing models, public–private partnerships, and global distribution initiatives may be valid and valuable for sustaining broadly just availability and provision of diagnostic and therapeutic developments, which may be contributory to de-limiting access to genetic assessments and therapies, thereby reducing inequities in ALS care.

## Conclusion

In recent years, there has been significant progress in both the understanding of the genetic architecture of ALS and the ability to target specific pathogenic variants using ASO technology, with Qalsody being the first example in the ALS field. Our expanding genetic knowledge has now blurred the historic classifications of familial and sporadic ALS and has moved to a focus on pathogenic variants and subsequent pathophysiological pathways.

With the emergence and increased accuracy of long-read sequencing technology it is now evident that all ALS patients should be offered whole-genome sequencing and that the current gaps in our understanding will continue to be filled as the genomes of ALS patients from varying ethnicities are sequenced and studied. ALS genetic architecture clearly differs between ethnic populations and the global efforts to establish improved, and population specific reference genomes will assist in the understanding of genetic drivers of disease in each specific patient group. This will not only enable a better understanding of the drivers of disease but will also allow the design of population specific treatments that encompass a true precision medicine approach.

Further, the ability to mine large data sets with bioinformatics tools has transformed the processing of both clinical and patient omics data and has also led to the improved stratification of ALS patient subgroups. While there is still much to uncover regarding how these pathophysiological sub-classifications may change over the course of the disease, this is a large step forward and will allow for targeted therapies to be designed and applied to the most relevant subgroups of patients.

In summary, to continue to ensure we can offer more targeted treatments for ALS patients, precision medicine principles must be applied throughout all phases of drug development. A comprehensive patient profiling approach will be key to ensure that *right drug is tested in the right patients*. Not only will a patient profiling workflow allow targeted therapies to be designed for the correct population of patients, but it will also assist in the identification of responders or non-responders that can be triaged onto alternative therapeutic options. As the field moves forward and ASOs enter platform trials, parallel in vitro testing will provide ongoing information about other novel therapies that might show improved efficacy in patient cells and leveraging patient long-read sequencing and omics data, one may be able to establish a digital or biomarker fingerprint of future patients to be recruited. Leveraging these latest advancements in the field, the effective translation of therapies for ALS patients will be achieved one patient sub-group at a time.

## Data Availability

No datasets were generated or analysed during the current study.
